# Polycystic ovaries and infertility: Our experience

**DOI:** 10.4103/0974-1208.44113

**Published:** 2008

**Authors:** Lavanya Rajashekar, Deepika Krishna, Madhuri Patil

**Affiliations:** Dr. Patil's Fertility and Endoscopy Clinic, No 1, Uma Admirality, First Floor, Bannerghatta Road, Bangalore, Karnataka-560 029, India

**Keywords:** Infertility, intrauterine insemination (IUI), *in vitro* fertilization (IVF), ovulation induction (OI), polycystic ovary syndrome (PCOS), pregnancy rate (PR), timed intercourse (TI), ultrasonography (USG)

## Abstract

**BACKGROUND:**

Polycystic ovary syndrome (PCOS) is one of the most common (15–20%) endocrine disorders in women of childbearing age. Although it is a major cause of infertility, its etiology remains unknown and its treatment difficult.

**AIM:**

To evaluate the incidence, treatment and outcome of patients with PCOS.

**DESIGN:**

Retrospective analysis.

**MATERIALS AND METHODS:**

PCOS patients (914 of the 1057) attending the outpatient department (OPD) from June 2003 to February 2008 were evaluated for this study. Of the 914 patients investigated, 814 came for treatment and these patients were studied for hormonal disturbances and their response to various modalities of treatment.

**RESULTS:**

Of the 2270 infertility patients, 46.50% (1057) had PCOS, out of these, 86.47% (914) were investigated and 77% (814) came for treatment. Our overall pregnancy rate was 48.40% (394/814). The pregnancy rate per cycle with timed intercourse (TI) was 44.77% (47/105), 17.09% (286/1673) with intrauterine insemination (IUI), 29.82% (51/171) with *in vitro* fertilization (IVF) and 22.22% (10/45) with frozen embryo transfer (FET). The maximum number of pregnancies (85.29%, 284/333) were achieved in the first three treatment cycles. The abortion rate was 19.01% (73/384) and the incidence of ectopic pregnancy was 5.47% (21/384). Complications seen were in the form of ovarian hyperstimulation (OHSS), retention cyst on day two and multiple pregnancies in 11.71% (228/1946) of the total treatment cycles.

**CONCLUSION:**

Most PCOS symptoms could be adequately controlled or eliminated with proper diagnosis and treatment. Thus, ovulation induction (OI) protocols and treatment modalities must be balanced for optimal results.

## INTRODUCTION

Polycystic ovary syndrome (PCOS) is a heterogeneous collection of signs and symptoms[1] which together, form a spectrum of the disorder with mild presentation in some and severe disturbance of reproduction, endocrine and metabolic function in others. The pathophysiology of PCOS appears to be multifactorial and polygenic. There are many extraovarian aspects to the pathophysiology of PCOS, yet ovarian dysfunction is the central aspect. The syndrome is surrounded by controversies regarding both its diagnosis and treatment.

The need to establish universally accepted diagnostic criteria led to the Rotterdam meeting[2] in 2003, but no optimal treatment was defined for infertile women with PCOS even then. The recognition of controversies surrounding the treatment of this enigmatic syndrome led to a second international workshop endorsed by the European Society for Human Reproduction and Embryology (ESHRE) and the American Society for Reproduction (ASRM), held in Thessaloniki, Greece in 2007.[3]

At one end of the spectrum, this heterogeneous disorder may present with a single finding of polycystic ovary (PCO) morphology as detected by ultrasonography (USG) [≥ 12 follicles 2–9 mm in diameter and/or increased ovarian volume (> 10 cm^3^)].[4] At the other end of the spectrum, symptoms such as obesity, hyperandrogenism, menstrual cycle disturbance and infertility may occur, either singly or in combination.

## MATERIALS AND METHODS

Polycystic ovary syndrome (PCOS) patients were selected on the basis of the Rotterdam criteria, (2003 ESHRE/ASHRM consensus), as per which the definition of PCOS requires the presence of two out of the following three criteria: 1. Oligo- and/or anovulation, 2. Hyperandrogenism (clinical and/or biochemical), 3. Polycystic ovaries on ultrasound, with the exclusion of other etiologies.[2]

We studied the incidence, hormonal profile, ovulation induction protocols, treatment modalities and outcome in patients with PCOS attending our Outpatient Department (OPD) from June 2003 to February 2008.

The basic work-up included a baseline ultrasound and measurement of the levels of follicle-stimulating hormone (FSH), luteinizing hormone (LH), prolactin and thyroid-stimulating hormone (TSH) on day two or three of the menstrual cycle. If TSH levels were abnormal, levels of free T4 (FT4) and thyroid peroxidase (TPO) was measured. FSH and LH levels of > 10 mIU/mL were considered to be elevated. Patients were treated for hypothyroidism if TSH was > 4.45 µIU/mL. All patients with prolactin levels > 25 ng/mL were treated for hyperprolactinemia. Progesterone levels were assessed on day 21 and an androgen profile: dehydroepiandrosterone sulfate (DHEAS) androstenedione (ASD), total and free testosterone was taken on day two of the menstrual cycle in patients who had hirsutism, acne or obesity. DHEAS levels of > 1500 ng/mL were targeted for treatment. ASD levels of > 2 ng/mL were considered to be elevated while levels > 0.8 ng/mL for total testosterone and 3.5 pg/mL for free testosterone were considered abnormal. A glucose tolerance test (GTT) and the measurement of fasting insulin (FI) levels were done in patients who were obese, those who were not able to lose weight despite exercise and diet control and those who did not respond to ovulation-inducing drugs. Patients who had an abnormal GTT or elevated FI levels (> 20 µU/mL) were started on metformin tablets at a dose of 1500 mg per day in divided doses. Tubal patency was documented either by hysterosalphingography (HSG) or laparoscopy. Semen analysis was done in all cases to rule out the male factor. If semen analysis revealed any abnormality (azoospermia, oligozoospermia, asthenozoospermia or teratozoospermia), the male partner was evaluated further.

As ours is a tertiary assisted reproductive technology (ART) centre, most patients who come to us have undergone either HSG or laparoscopy for tubal assessment. Patients who had undergone HSG or laparoscopy, but who did not conceive or respond after 4–6 cycles of ovulation induction (OI) with either timed intercourse (TI) or intrauterine insemination (IUI) were counseled either for laparoscopic ovarian drilling (LOD) or *in vitro* fertilization (IVF) / intracytoplasmic sperm injection (ICSI). A hysteroscopy was done only if patients opted for IVF, hysterolaparoscopy being done if these patients desired to continue with IUI. Laparoscopy was done before OI in 21 patients as they either had endometriosis or HSG showed a tubal block.

Lifestyle modification was the first line of therapy in obese patients. Drugs used for OI were: 1. clomiphene citrate (CC), 2. letrozole, 3. CC/letrozole + gonadotrophins (GT), 4. GT, 5. Gonadotrophin-releasing hormone (GnRH) agonist + GT.

Either CC or letrozole was given to induce ovulation for timed intercourse. The cycles were monitored by ultrasound for follicular growth and a postcoital test (PCT) was done 36 h after the hCG trigger which was given at a follicular size of 20–22 mm. Patients targeted for IUI, underwent ovulation induction with CC, letrazole, a combination of CC/Letrazole + GT or a GnRh agonist in a short protocol (day 2 until the day of hCG administration) + GT. GnRH agonists were used in the long protocol with GT for all ART cycles. The GT used and the dose were determined based on day two LH levels, the response in previous GT stimulation cycles and any previous history of (H/O) ovarian hyperstimulation syndrome (OHSS). In patients with elevated LH levels, excessive response in previous GT cycles and previous H/O OHSS were given recombinant (REC) FSH so that the dose could be reduced to even 25 IU. Depending on the hormonal levels, previous response to ovulation induction drugs and the presence or absence of hyperandrogenemia and obesity, the other patients were given either pure FSH, FSH, hMG or a combination of FSH + hMG. Follicular growth and endometrial thickness were monitored by ultrasound. A baseline scan was done on day two for all patients before they were started on any OI drugs. Patients on CC/letrozole and those on GT were monitored daily from days nine and five onwards respectively. Follicles were tracked on alternate days until they reached a size of 13–14 mm, after which USG was done daily. Estradiol levels were measured on days 5, 9 and on the day of human chorionic gonadotrophin (hCG) administration in all IVF cycles. In TI and IUI cycles, estradiol levels were measured only if there was no or excessive response with the aim of increasing or decreasing the dose of GT. LH levels were measured only in those patients who were found to have hyperechoic endometrium during USG monitoring of follicular growth. Monitoring was not stopped even if patients did not ovulate by day 15 and was continued until day 21 of the cycle. Monitoring and treatment was continued if a dominant follicle > 14 mm in size was seen on day 21. Tablet Dexamethasone 0.5 mg was given at 10 P.M. to all patients with elevated DHEAS levels from day two to day eleven of the OI cycle.

Treatment modalities offered to our patients undergoing OI were either TI with a postcoital test (PCT), IUI or ART. The indications for 171 ART cycles (IVF/ICSI) were (i) failed IUI (44), (ii) tubal factor (21), (iii) male factor (58), (iv) endometriosis (16) and (v) presence of > six dominant follicles in the IUI cycle (32).

A long GnRH agonist protocol (250 µg given subcutaneously twice daily from day 21 of the previous cycle until day two and once a day thereafter) was used with GT in 139 ART cycles. Patients who underwent ART due to the presence of > six dominant follicles in an IUI cycle were targeted for treatment with a short GnRH agonist protocol with GT. Seven patients underwent cycle cancellation while patients who did not agree for IVF or cycle cancellation, underwent IUI, multiple pregnancies were seen in such patients.

Frozen embryo transfer (FET) was done either in a natural (if the patient ovulated naturally) or hormonal replacement therapy (HRT) cycle.

All patients were given luteal phase support with Tablet Dydrogesterone 10 mg twice a day / progesterone vaginal pessaries 400 mg twice a day / progesterone Injection 100 mg IM daily for 15 days. Beta hCG was done 15 days after ovulation and if positive, progesterone was continued. Clinical pregnancy was documented 20 days after ovulation. Progesterone was continued for 12 weeks of pregnancy in the presence of a viable intrauterine pregnancy.

## RESULTS

**Figure 1:**
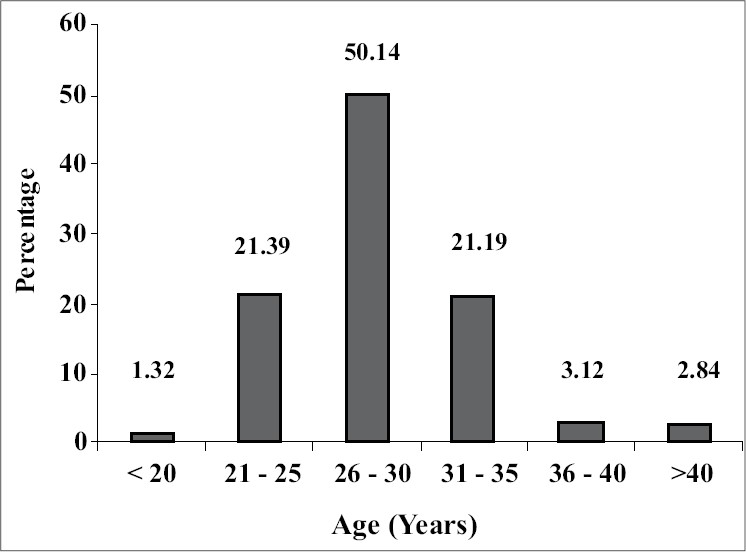
Age distribution

Of all infertility patients (2270) seen in the last five years, 46.50% (1057) were PCOS patients, out of which 86.47% (914) were investigated and 77% (814) came for treatment. Eight hundred and ninety-six (84.76%) of these patients had primary infertility, most of whom 71.53% (752) were in the age group of 21–30 years [Figure 1]. Obesity and excess androgen(s) were seen in 48.72% (515) and 73.51% (777) of all patients respectively. Whereas 41.06% (434) patients had normal cycles, menstrual disturbance was seen in 51.84% (548) patients in the form of oligomenorrhea or amenorrhea and 7.10% (75) of the patients had menstruation only with progesterone withdrawal.

A minority 5.25% (48) of the patients had elevated follicle stimulating hormone (FSH) levels. Luteinizing hormone (LH) levels were elevated in only 17.18% (157) of the patients whereas 11.49% (105) had elevated prolactin levels and were treated with tablet cabergolin 0.5 mg once a week for six weeks after which prolactin levels decreased. Elevated levels of DHEAS, ASD and testosterone were observed in 65, 54.27 and 44.64% of the patients respectively. Hypothyroidism was seen in 20.46% (187) of the patients who were treated with tablet eltroxin or tablet thyronorm. Hyperinsulinemia was seen in 31.29% (286) of the patients who were treated with tablet metformin at a dose of 500 mg three times a day.

Along with PCOS, 19.69% (180/914) of the patients had associated male factors with azoospermia in 16.11% (29/180) and oligospermia / asthenospermia in 83.89% (151/180) of the patients. Other etiological factors contributing to infertility, such as endometriosis, tubal factors, pelvic inflammatory disease (PID), cervical factors, intrauterine adhesion or uterine anomalies were seen in 18.71% (171/914) patients.

The pregnancy rates with and without LOD are depicted in Table 1. Only one patient conceived immediately after LOD without OI and 128 patients underwent OI after LOD as they did not ovulate spontaneously.

**Table 1:** Treatment modalities and results

Treatment modality	Total	%	No. of pregnancies	*P* value
Laparoscopic	10	12.28	1 (10)	0.015*
Laparoscopic and medical	128	15.72	98 (76.56)	0.277
Medical	676	83	295 (43.64)	<0.001**
Total	814	-	394 (48.40)	-

* Statistically significant

** Statistically highly significant, Figures in parentheses are in percentages

Table 2 highlights the different OI protocols for IUI with respective pregnancy rates in 1673 IUI cycles. 12.74% (85/667) conceived with CC, 17.08% (115/673) conceived with letrozole, 28.79% (19/66) conceived with CC/letrozole + hMG, 27.78% (10/36) conceived with gonadotrophins and 24.67% (57/231) conceived with the combination of a GnRH agonist + GT. The pregnancy rates for IUI were significantly higher when CC 200 mg and GnRH agonist short protocol with GTs were used for OI, which is statistically significant as evident from the P values mentioned in Table 2. As seen in Table 2A, pregnancy rates were significantly higher with a short protocol combining GnRHa and GT as compared to GT alone or GT in combination with CC/Letrazole (*P* = 0.003). Out of 1673 IUI cycles, 56.48% (945/1673) patients ovulated within 16 days of which 171 (18.09%) conceived, 7.53% (126/1673) did not ovulate and 35.98% (602/1673) patients ovulated after 17 days of stimulation. Of these 602 patients who ovulated after 17 days of stimulation, 30.42% (509) ovulated between days 17–22 (of these 13.56% (69) conceived), 4.45% (75) ovulated between 23–30 days (of these, 34.67% (26) conceived) and 1.06% (18) ovulated after 30 days (out of which 27.78% (5) conceived). Maximal pregnancy rates (85.29% (284/333)) were obtained in the first three treatment cycles of TI or IUI, irrespective of the OI protocol used [Table 3].

**Table 2:** Ovulation induction protocol for intrauterine insemination

Drug	No. of cycles	Pregnancies	*P* value
CC‡ 100 mg	72	11 (15.28)	0.682
CC 150 mg	35	6 (17.14)	0.995
CC 200 mg	560	68 (12.14)	0.002**
Letroz 2.5 mg	77	10 (12.99)	0.338
Letroz 5 mg	176	36 (20.45)	0.239
Letroz 7.5 mg	420	69 (16.43)	0.715
CC/Letroz + hMG§	66	19 (28.79)	0.018*
hMG	27	6 (22.22)	0.479
FSH* + hMG	9	4 (44.44)	0.029*
GnRHa** + FSH	43	7 (16.27)	0.885
GnRHa + REC‡‡.FSH	15	5 (33.33)	0.095+
GnRHa + hMG	160	40 (25)	0.008**
GnRHa + FSH + hMG	13	5 (38.46)	0.041*
Total cycles	1673	286 (17.1)	-

Figures in parentheses are in percentages

‡ = Clomiphene Citrate

§ = Human Menopausal Gonadotrophin

* = Follicle Stimulating Hormone

** = Gonadotrophin Releasing Hormone Agonist (short protocol)

‡‡ = Recombinant

* Statistically significant

** Statistically highly significant

**Table 2A:** Comparative pregnancy rates with different gonadotrophin protocols for intrauterine insemination

Drug	No. of cycles	Pregnancies	*P* value
CC‡/letrozole +hMG	66	19 (28.8)	0.0166*
GT‖	36	10 (27.8)	0.0882
GnRHa** + GT	231	57 (24.6)	0.003**
	1673	286 (17.1)	

* Statistically significant

** Statistically highly significant

‡ Clomiphene Citrate

§ - Human Menopausal Gonadotrophin

* - Follicle-stimulating Hormone

‖ - Gonadotrophin

** -Gonadotrophin-releasing Hormone Agonist (short protocol)

‡‡ - Recombinant, Figures in parentheses are in percentages

**Table 3:** Number of cycles required for conception with intrauterine insemination/timed intercourse

Therapy	Total pregnancy	1	2	3	4	5	> 6
CC‡ + TI†	27	12	10	5	-	-	-
CC + IUI	85	45	15	14	5	4	2
Letrozole + TI	20	7	8	5	-	-	-
Letrozole + IUI	115	47	36	15	15	1	1
CC/Letrozole + GT‖ + IUI	19	6	7	3	3	-	-
hMG§ + IUI	6	1	2	3	-	-	-
GnRHa**+ hMG+FSH*+IUI	2	1	1	-	-	-	-
GnRHa + FSH + IUI	14	8	2	3	1	-	-
GnRHa + hMG + IUI	45	7	9	12	8	7	2
Total	333	134 (40.24)	90 (27.03)	60 (18.02)	32 (9.61)	12 (3.60)	5 (1.50)

‡ - Clomiphene Citrate

‖ Gonadotrophin

§ - Human Menopausal Gonadotrophin

* - Follicle-stimulating Hormone

** - Gonadotrophin-releasing Hormone Agonist (short protocol) Figures in parentheses are in percentages

Controlled OI protocols are highlighted in Table 4 for IVF/ICSI with the pregnancy rates for each protocol. Out of the 171 cycles, 34 (19.88%) only had a biochemical pregnancy and 51 (29.82%) had a clinical pregnancy. The pregnancy rate was the same with different OI protocols as evident by the *P* value, but the *P* value was statistically significant for the long protocol in comparison to the short one. Of the 51 clinical pregnancies we observed with ART, 88.24% (45) conceived in the first cycle, 9.80% (5) conceived in the second cycle and 1.96% (1) conceived in the third cycle.

**Table 4:** Ovulation induction Protocols for *in vitro* fertilization/ intracytoplasmic sperm injection

Protocol	Total cycle	Biochemical pregnancy	Clinical pregnancy	*P* value
GnRHa** Long + FSH*	1	0	0	-
GnRHa Long + REC‡‡. FSH	10	2	3 (30)	1.000
GnRHa Long + FSH + hMG§	128	29	34 (26.56)	0.055
GnRHa Short + hMG	4	1	1 (25)	1.000
GnRHa Short + FSH + hMG	3	0	2 (66.67)	0.514
GnRHa Short + REC. FSH	23	2	11 (47.83)	0.049
GnRHa Short + FSH	2	0	0	-
Total	171	34	51 (29.82)	

χ^2^=4.424

*P* value = 0.035 for GnRH agonist long vs short protocol

§ - Human Menopausal Gonadotrophin

* - Follicle-stimulating Hormone

** - Gonadotrophin-releasing Hormone Agonist

‡‡ - Recombinant, Figures in parentheses are in percentages

**Figure 2:**
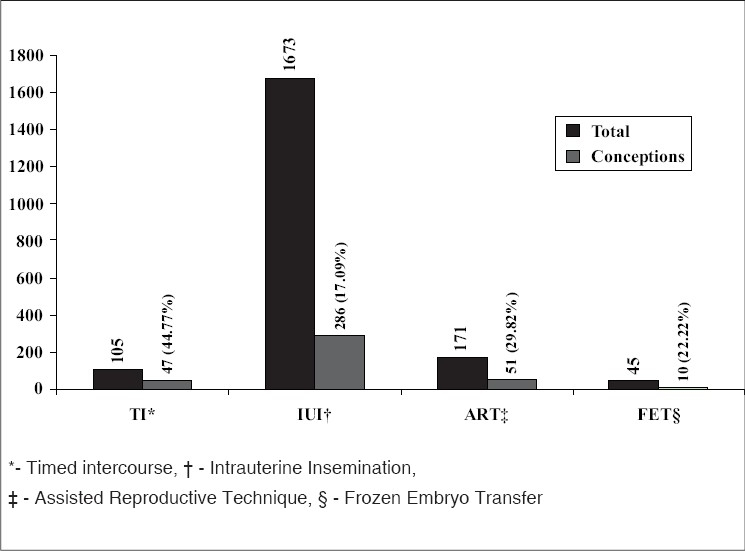
Treatment modality and pregnancy rate

**Figure 3:**
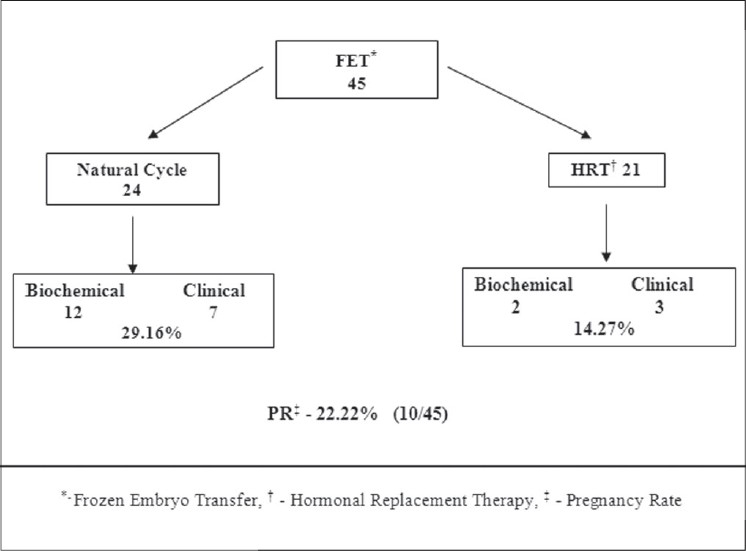
Frozen embryo transfer

Clinical pregnancy rate per treated cycle [Figure 2] was 44.77% (47/105) with TI, 17.09% (286/1673) with IUI, 29.82% (51/171) with fresh IVF / intracytoplasmic sperm injection (ICSI) and 22.22% (10/45) with FET. Of the 45 FET cycles, 24 were in a natural cycle and 21 in an HRT cycle; the results of FET cycles are highlighted in Figure 3.

Table 5 shows the overall pregnancy outcome with various treatment modalities. Of the total 384 pregnancies, 19.01% (73) had first or second trimester abortion, 5.47% (21) had ectopic pregnancies and the remaining 290 carried on to the second trimester. The outcome after the second trimester was not known for all patients as they were referred to an obstetrician after 16 weeks.

**Table 5:** Analysis of pregnancy outcome

Treatment	Total	Spontaneous abortion		Ectopic			
			Continued after II trimester			

		1^st^ trimester	2^nd^ trimester		n	%	*P* value
CC	109	16	3	6	84	77.1	0.697
Letrozole	138	21	2	6	109	78.9	0.353
CC/Letrozole + hMG	19	5	-	1	13	68.4	0.471
GT	10	1	-	3	6	60.0	0.254
GnRHa + GT	108	24	1	5	78	72.2	0.363
Total	384	67	6	21	290	75.5	-
Percentage		17.45	1.56	5.47		75.52	

‡ Clomiphene citrate

‖ - Gonadotrophin

§ - Human menopausal gonadotrophin

** - Gonadotrophin-releasing hormone agonist

Complications were seen in the form of OHSS in 228/1946 cycles with an incidence of 11.71%. Ovarian cysts were observed on day 2 of the next cycle in 3.49% (68) of the patients and multiple pregnancies were seen in 2.87% (56) of the patients with different OI protocols [Table 6]. The incidence of multiple pregnancies was significantly high in the IUI cycle as compared to the ART cycle (71.43 *vs* 28.57%) which was statistically significant (*P* < 0.001) [Table 7]. This is because the number of oocytes ovulated, fertilized and implanted could not be controlled in IUI cycles. Six patients did not agree for IVF or cycle cancellation and wanted to continue with IUI even after being counselled regarding the risk of OHSS and multiple pregnancies. These are the patients who had high-order multiple pregnancies in our series. The incidence of high-order multiple pregnancies was low with ART at our center, as we typically do not transfer > 2 embryos. One triplet in the IVF cycle was due to monozygotic twinning.

**Table 6:** Complications of ovulation induction in intrauterine insemination/assisted reproductive technique

Therapy	Total	OHSS	Ovarian cyst	Multiple pregnancy
CC§	34	14	15	5
Letrozole	28	11	13	4
CC/Letroz + GT‖	39	13	18	8
GT	36	16	10	10
GnRHa**+ GT	91	50	12	29
Total (1946 cycles)	228 (11.7)	104 (5.34)	68 (3.49)	56 (2.87)

§ -Clomiphene citrate

‖ -Gonadotrophin

** -Gonadotrophin-releasing hormone agonist, Figures in parentheses are in percentages

**Table 7:** Multiple pregnancies

Order	Total	IUI¶	ART††
Twins	46	31	15
Triplets	5	4	1
Quadruplets	2	2	-
Quintuplets	1	1	-
Septuplets	1	1	-
Nonuplets	1	1	-
Total	56	40	16
		(71.43%)	(28.57%)

*P* <0.001, Statistically significant

¶ - Intrauterine insemination

†† - Assisted reproductive technique

## DISCUSSION

According to the Rotterdam definition, any case of PCOS should be categorized, based on the presence or absence of insulin resistance and whether the women are asymptomatic or symptomatic. Lifestyle changes are critically important for successful management and long-term health. Hyperandrogenism is optimally dealt with by reducing insulin drive to the ovary, with measures such as exercise and diet modification. The aim of management should be unifollicular ovulation and minimizing the risk of OHSS and multiple pregnancies.

In our series, preconception counseling was provided to all patients before any intervention was initiated and the importance of lifestyle modifications, especially weight reduction and exercise in overweight women, smoking and alcohol consumption cessation was emphasized, as was the need to identify risk factors for reproductive failure and their correction prior to treatment initiation.

Today, CC still remains the first line of treatment in PCOS although aromatase inhibitors are an effective, inexpensive and safe alternative to CC. Insulin sensitizers have proven to be beneficial only in obese women (BMI > 35 kg/m^2^ ) with glucose intolerance. CC and letrozole resistance or failure was treated with second line intervention in the form of either exogenous gonadotrophins or laparoscopic ovarian surgery (LOS). *In vitro* fertilization was the third line of treatment.[3]

It is well known that obesity is associated with anovulation, pregnancy loss and late pregnancy complications. Obesity is common in women with PCOS and is linked to failure or delayed response to the various treatment protocols offered for ovulation induction. These changes have been reported with a weight loss as small as 5% of the initial weight.[5] Lifestyle modifications were recommended to all our obese patients along with ovulation induction, as it is associated with spontaneous ovulation and higher pregnancy rates.[3]

The use of dexamethasone as an adjunct with OI drugs in patients with elevated dehydroepiandrosterone sulfate (DHEAS) levels, suppresses the adrenal androgen secretion and induces responsiveness to OI in previous nonresponders.[6]

Elevated LH levels (about 95^th^ percentile of the normal) can be observed in approximately 40–60% of PCOS patients.[7] In our study, only 17.18% (157) of patients had elevated LH levels. Tonic LH secretion in the follicular phase affects oocytes by the early resumption of meiosis and premature oocyte maturation with ovulation of the prematurely mature egg will result in either an inability to be fertilized or a miscarriage if fertilization occurs.[8] Balen *et al.* reported that PCOS had a miscarriage rate of 36% when compared with 24% in women with normal ovaries, which is also consistent with our findings wherein 35/75 who aborted, were found to have elevated LH levels.[9]

Laparoscopic ovarian diathermy (LOD) has taken the place of wedge resection of the ovaries and carries a reduced risk of multiple pregnancies compared with gonadotrophin therapy in the treatment of CC-resistant cases.[10] Premature ovarian failure is a concern with ovarian drilling, especially when a large number of punctures are made (> 4–6).[3] In our study, three such cases were detected, probably in those who had > 6–8 punctures done. This led Armar *et al*. to develop what has become the most favored strategy of minimizing the number of diathermy points to four; each for 4 seconds at 40 W at a depth of 4 mm, using a monopolar needle.[11] This also correlates with our study where the *P* value is statistically significant (< 0.001).

Ovulation is normally restored with clomiphene citrate in approximately 80% of the patients, with a pregnancy rate of 35–40%, out of which, 75% of the pregnancies were achieved within the first three treatment cycles.[12] In our study, we had a 13.19% (88/667cycles) pregnancy rate with different doses of CC, 84.09% (74/88) of the pregnancies being achieved within the first three treatment cycles; 10.9% (73/667) of the cases had CC resistance.

Letrozole, an aromatase inhibitor, has shown to be effective in inducing ovulation and pregnancies in women with anovulatory PCOS and in patients with CC resistance or failure.[13] It also improves the ovarian response to FSH in poor responders.[14] We had better results with letrozole as compared with CC [17.09% (115/673 cycles) *vs* 12.74% (85/667 cycles) respectively]. A GnRH agonist was used in the short protocol with GT in most of our IUI cases. We used a GnRH agonist in most IUI cycles for the following two reasons: 1. The use of GT alone could result in a premature LH surge with elevated progesterone levels in the late follicular phase, which could then compromise the oocyte quality and pregnancy rate of the IUI cycle, 2. The problem of premature rupture of follicles and premature lutenization should not occur as a result of endogenous LH surge if an IUI cycle needs to be converted into an IVF cycle. One problem seen with the use of a GnRH agonist with GT in a short protocol, if the dose of GT is not kept low for IUI cycles, is multifollicular development with its complications of OHSS and multiple pregnancies.

Currently, the most standard protocol for ART is a long desensitization GnRH agonist protocol along with FSH.[3] Administration of FSH alone is a conceptually better option and tends to be associated with a significantly higher pregnancy rate according to the Cochrane Database Systemic review published in 2000. Later in 2001, the Cochrane database again published that there was no significant differences in ovulation rate, TI, miscarriage rate, multiple pregnancy rate or OHSS whether one used REC FSH, highly purified or urinary FSH or hMG for ovulation induction. Thus, due to these varied opinions, the optimal stimulation protocol is still under debate. A GnRH agonist eliminates a premature LH surge and any subsequent premature luteinization and reduces the relatively low pregnancy rates and high miscarriage rates witnessed in PCOS patients[15] with better quality of eggs.[16] In our study, the overall pregnancy rate was significantly higher in gonadotrophin cycles compared to CC / Letrozole, which was itself statistically significant (25.82% {86/333} *vs* 14.93% {200/1340}) Gonadotrophin therapy may be superior in achieving higher pregnancy rates compared to CC or letrozole, but it is expensive and there is a significantly higher risk of ovarian hyperstimulation and multiple pregnancies,[10] which can also be seen in our study.

The most dreaded complication of controlled ovarian stimulation is the occurrence of OHSS.[3] Multiple pregnancies are very common with OI in PCOS, be it CC, letrozole or GT, though they are higher with GT. One could reduce the number of multiple pregnancies by using softer stimulation protocols and restricting the number of embryos transferred to one or two in ART. Hamilton-Fairley and Franks reported a mean multiple pregnancy rate of 34% and a severe OHSS rate of 4.6%.[17] Scheneker and Weinstein[18] reported mild, moderate and severe hyperstimulation occurring in 8.4–23%, 6–7% and 0.8–2% of treatment cycles, respectively. The incidence of OHSS in our study was 5.34% (104/1946 cycles) whereas that of multiple pregnancies was 2.87% (56/1946 cycles), which is comparable with other publications.

It is essential that follicular maturation occur in an environment free from elevated LH levels to enhance the fertilization and pregnancy rates. Timed intercourse and IUI should remain the basic treatment modality for these patients, unless there are any of the other coexisting factors for infertility, which warrant ART. IVF/ICSI should be offered to only those who are refractory to conventional treatment modalities.

## CONCLUSION

Lifestyle modifications involving both diet and exercise, helped in improving the ovulation and pregnancy rates in our obese patients. In our study, we showed that letrozole is as effective as CC in inducing ovulation, with a slightly higher pregnancy rate. Thus, letrozole could be used as a first line of therapy with fewer mature follicles and a resultant decreased incidence of OHSS and multiple pregnancies. Letrozole also does not have any antiestrogenic effects but still results in a better pregnancy rate. Metformin was effective only in those patients who had high fasting insulin levels and glucose intolerance and were able to lose weight only when on metformin. The pregnancy rate is higher when ovulation induction drugs are used along with metformin as compared to metformin alone. A low-dose (37.5 IU) step-up protocol of GT is efficient in inducing monofollicular development with a good pregnancy rate, though a greater number of days is required for the follicle to reach the ovulatory diameter. Strict cycle cancellation criteria should be followed to prevent multiple pregnancies and OHSS. This is in view of the multiple pregnancies we observed in our series in those women who did not agree to cycle cancellation or conversion to an IVF cycle. Thus, it is better to use a short GnRH agonist protocol with GT in IUI cycles, as it becomes easy to convert these patients into an IVF cycle without the risk of a premature LH surge. The only disadvantage is that we cannot use GnRH agonist as a trigger in patients who have a risk of developing OHSS. IVF remains a good option in PCOS patients who overrespond or do not conceive after 4–5 cycles of IUI. It helps in identifying the oocyte quality which may be impaired in patients with PCOS, minimizing the risk of multiple pregnancies by transferring a small number of embryos and preventing or reducing the intensity of OHSS by freezing all embryos for transfer in future cycles.
